# Usefulness of repair using Hem-o-lok™ for peritoneal tear as a complication of totally extraperitoneal repair: Case series

**DOI:** 10.1016/j.amsu.2019.11.011

**Published:** 2019-11-23

**Authors:** Toshikatsu Nitta, Jun Kataoka, Masato Ohta, Kensuke Fujii, Youko Takashima, Takashi Ishibashi

**Affiliations:** Division of Surgery Gastroenterological Center, Medico Shunju Shiroyama Hospital, Osaka, Japan

**Keywords:** Hem-o-lok, Peritoneal tear, Totally extraperitoneal repair, CG, Combination of Hem-o-lok stapling group, PT, peritoneal tear, SG, suturing group, TAPP, transabdominally preperitoneal repair, TEP, extraperitoneal repair

## Abstract

**Introduction:**

Peritoneal tear (PT) is a frequent intraoperative event during totally extraperitoneal repair (TEP). We aimed to introduce our surgical technique for PT during TEP to avoid the more difficult TEP procedure.Methods

One surgeon with 10 years of experience performed our TEP method in 147 TEP cases from January 2012 to June 2019. We investigated the repair time of each repair technique using endoscopic suturing (suturing group, SG) and endoscopic Hem-o-lok stapling (CG).

**Results:**

Twenty-three (15.6%) PT cases occurred as TEP complication. The mean repair times (with standard deviation) of the PT were 16.2 ± 13 and 7.6 ± 7.0 min in the SG and CG, respectively, indicating a significant difference (P = 0.043). The repair time of the PT using Hem-o-lok (Teleflex, Wayne, PA, USA) stapling was shorter than that using endoscopic suturing, which was significantly different despite the length of the PT.

**Conclusion:**

Hem-o-lok stapling is feasible in case of PT during TEP.

## Highlights

• The peritoneal tear repair time when using Hem-o-lok stapling was shorter than that using endoscopic suturing.
• Hem-o-lok stapling is very effective for peritoneal tear during extraperitoneal repair.
• The technique is simple and can possibly replace metal stapling.

## 1 Introduction

Laparoscopic surgery became a widely accepted surgical treatment for inguinal hernia recently. It offers less postoperative pain, faster recovery, and lower recurrence rate than open surgery [1,2]. The laparoscopic approach for inguinal hernia repair can be performed through transabdominally preperitoneal (TAPP) or totally extraperitoneal (TEP) approach. The European Hernia Society [3] recommended TEP for endoscopic inguinal hernia operations. However, TEP is considered a technically difficult procedure, with a more demanding learning curve, due to the unfamiliar visualization of the inguinal anatomy [4].

In Japan, endoscopic procedures were performed in 59,888 patients (20.2%), and these procedures included TAPP and TEP repair in 41,699 (14.1%) and 18,219 patients (6.2%), respectively [5]. Many surgeons in Japan choose TAPP because the surgical anatomy is easier to understand with TAPP than with TEP. General surgeons are not usually accustomed to the TEP field. The working space in TEP is narrower and limited, which hinders the mobility of the surgical equipment.

Peritoneal tear (PT) is a frequent intraoperative event during TEP and is recognized as a common and major complication in TEP [6,7]. Occurrence of PT during TEP results in pneumoperitoneum and loss of extraperitoneal space [8]. Furthermore, the laparoscopic suture technique is difficult and troublesome in narrower space, compared with that of TAPP, in PT repair.

Since 2012, we used TEP as a first choice in our hospital. More than 300 cases have been performed safely since its first introduction. We aimed to report our technique for repairing PT during TEP and thus avoid the more difficult TEP procedure.

## 2 Methods

### 2.1 Registration and ethics

Written informed consent was obtained from the patients for the inclusion of their information in this study.

### 2.2 Reporting guideline

This case has been reported in line with the SCARE criteria [8], and the surgical technique was based on the PROCESS guidelines [9].

### 2.3 Methods

One surgeon under 10 years of experience performed the TEP technique in 147 cases from January 2012 to June 2019. The incidence of PT as a complication was investigated. We investigated the repair time of each repair technique using endoscopic suturing (suturing group, SG) and endoscopic Hem-o-lok stapling (Hem-o-lok group, CG). Both groups were compared in terms of patient's age during surgery, sex, hernia type (bilateral or unilateral), mean operative time, mean length of the PT, and mean repair time of the PT (Table 1).

**Table 1:** Characteristics of patients in whom peritoneal tear occurred.

	Suturing group (n = 15)	Combination with Hem-o-lok group (n = 8)	P-value
Age (years)	64 ± 9.4	65 ± 16	**0.42**
Sex	male	male	–
Hernia type (indirect/direct)	15 : 0	7 : 1	–
Bilateral/unilateral	9 : 6	6 : 2	–
Mean operative time (min)	192 ± 60	171 ± 60	**0.22**
Mean length of peritoneal tear (mm)	30 ± 15	35 ± 21	**0.26**
Mean repair time of peritoneal tear (min)	16.2 ± 13	7.6 ± 7	**0.043**

Values are presented as mean ± standard deviation.

Suturing group (n = 15) Combination with Hem-o-lok group (n = 8).

### 2.4 Surgical technique

**Fig. 1:**
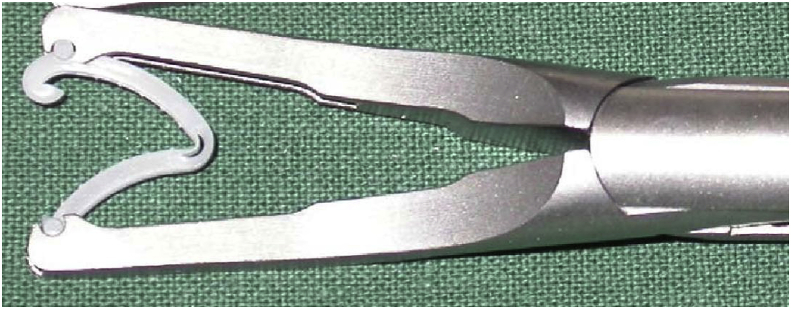
Hem-o-lok stapling.

**Fig. 2:**
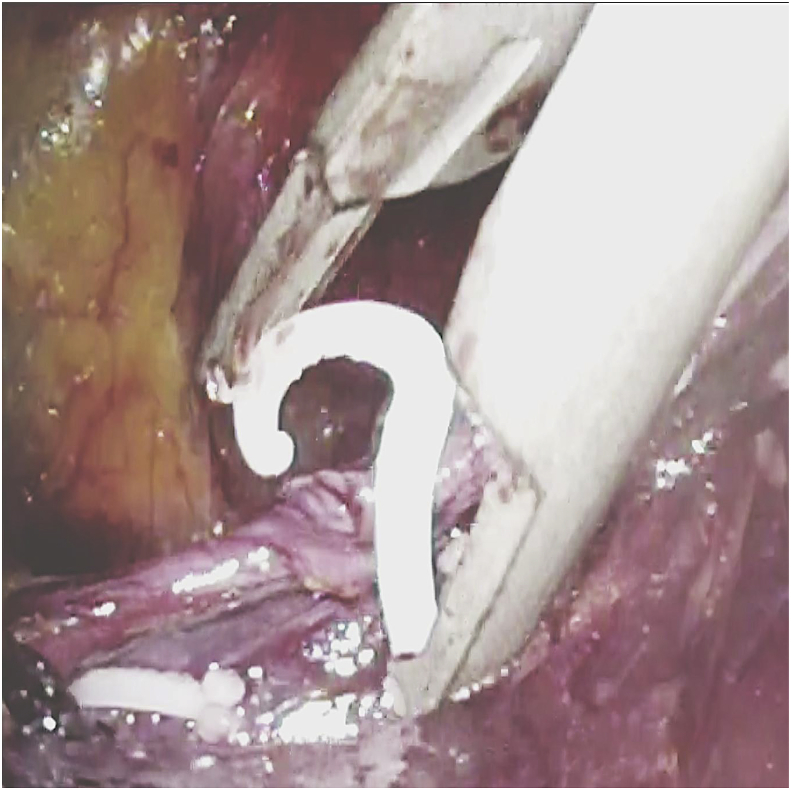
Peritoneal tear being closed by Hem-o-lok stapling.

**Fig. 3:**
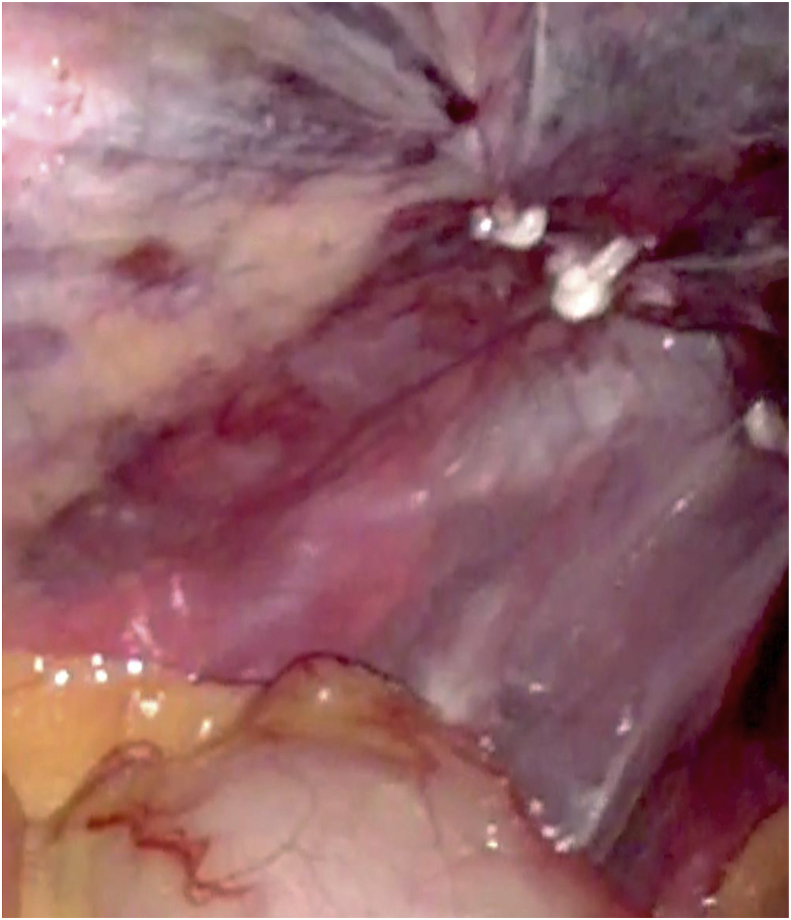
Repair using Hem-o-lok stapling for peritoneal tear.

Briefly, in our surgical technique for TEP [10], a port was placed 12 mm below the umbilicus at the midline. Two 5-mm ports were inserted in the midline. We determined first whether bilateral inguinal hernia was present through the intra-abdominal scope via laparoscopic examination. Then, we dissected the space of Retzius inside the epigastric arteriovenous pedicle. Dissection was performed through a sub-umbilical incision without a balloon. Cord structures were isolated as part of parietalization. We located and traced the peritoneal edge, as this layer should be divided. Dissecting and separating the peritoneal edge is easier; however, this layer is thin and fragile, causing PT [11]. PT repair requires laparoscopically repair of the peritoneum through suturing and stapling (Hem-o-lok™; Teleflex) (Fig. 1) [12] and suture loop ligation (Surgitie™; Covidien). We performed this quick technique using Hem-o-lok (Weck Closure Systems, Research Triangle Park, NC, USA) for PT (Fig. 2). Subsequently, 3DMAX™ (3D Mesh; Brad) was placed in the preperitoneal space, and tacking was accomplished by AbsorbaTack™ (Covidien). We reviewed the results of the operation laparoscopically and determined whether the repair was satisfactory (Fig. 3).

### 2.5 Statistical analysis

Statistical analysis was performed with the Mann-Whitney *U* test. Statistical differences were considered significant at P < 0.05.

## 3 Results

A total of 147 patients underwent our TEP method performed by only one surgeon. All patients were men aged 19–82 years. Hernia type was mainly indirect hernia (95.7%), and one patient had a case of direct hernia. PT tends to occur more often in bilateral hernia (65.2%).

Of these patients, 23 PT (15.6%) cases had a complication. The mean operative times of our TEP technique with and without PT were 149.7 and 184.4 min in 124 and 23 cases, respectively. No significant difference was found in the occurrence of PT between the two groups (P = 0.052).

The closure of the PT was done with endoscopic suturing combined with suture loop ligation (SG, 15 cases) and stapling (CG, 8 cases). No significant difference in operative time or mean length of the PT was noted between the two groups (P = 0.22 and P = 0.26, respectively).

The mean repair times (with standard deviation) of the PT were 16.2 ± 13 and 7.6 ± 7.0SD min in the SG and CG, respectively, and a significant difference was observed between the two groups (P = 0.043).

## 4 Discussion

Laparoscopic hernioplasty is commonly performed in Japan. However, the posterior approach, such as TEP, is not widely used, and only about 10% of inguinal herniorrhaphy cases in Japan are treated using this method because it involves a complicated anatomy [13]. Many surgeons are not familiar with the unusual, complex anatomy of the extraperitoneal space; thus, the occurrence of PT during TEP results in pneumoperitoneum and quick loss of extraperitoneal space [11].

Kugel posterior herniorrhaphy was introduced in 1999 [14] and is known as a reasonable method. However, this approach is associated with a steep learning curve and a high recurrence rate during the early learning time [15,16]. As a result, the posterior approach is not widely used, and only about 10% of inguinal herniorrhaphy in Japan utilizes this method because of the complicated anatomy involved [10,13].

The laparoscopic approach (posterior approach) adapts the advantages of Kugel hernioplasty, making it possible to perform at a new layer even if inguinal hernia recurs after the anterior approach, thus producing a high level of completion [10].

Moreover, reaching the extraperitoneal space is difficult. PT is a common complication of TEP, and the incidence of intraoperative PT ranges from 10% to 64% [11], which prolongs the operative time. In this study, the incidence of PT was 15.6%, and the mean operative time of TEP increased once PT occurred. Although no significant difference was found, the P-value was 0.052, which approached significance; however, the sample size is too small, but it is possible that this is related to the prolonged operative time.

The repair time of PT was shorter, if the total operative time is considered. This is because the real repair time was only approximately 10 min, but the preparation for repair takes a long time. The preparation includes the search for the position of the PT to be repaired, carrying the needle to the narrow working space, and others. The presence of PT might prolong the duration of operation, making it one of the key factors to consider when performing TEP.

Lau et al. [11] compared the operative time and postoperative morbidity among endoscopic metal stapling, endoscopic suturing, and pre-tied suture loop ligation. Endoscopic metal stapling enables a shorter operative time in case of PT. Endoscopic stapling and pre-tied suture loop ligation are safe and quick techniques for the closure of PT during TEP. From our data (Table 1), the total number of PT did not show significant difference, and the repair time of the PT using Hem-o-lok was shorter than that when using endoscopic suturing. However, we did not find any significant difference in the total operative time between the two groups, even if the repair time using Hem-o-lok showed significant difference despite of the length of the PT. PT is classified based on its length, short and long, and these types are further subdivided into single and multiple types (Table 2). Our quick technique appeared to be effective, but short and single, multiple type is possible to be very effective.

**Table 2:** Types of peritoneal tear.

Single type	Short type
	Long type
Multiple type	Short type
	Long type

There was no complication after our technique during the follow-up.

Many surgeons perform the technique using endoscopic metal stapling to treat PT, but this study is the first to report on the use of Hem-o-lok for PT. Our technique, which uses endoscopic Hem-o-lok stapling, had almost the same outcomes as those of endoscopic metal stapling and is unique and safe for PT.

With the Weck® Hem-o-lok® Polymer Ligation System, surgeons can use a secure polymer clips ligation modality [12]. The Weck® Hem-o-lok® Polymer Ligation System consists of permanent nonabsorbable, nonconductive polymer clips that are secure and easy to use in surgery [12]. The clips have a distal locking mechanism and grooves that enable them to securely ligate 2- to 16-mm vessels and tissue [12].

Hem-o-lok is not detectable on X-ray and computed tomography, unlike metal clips. This is very important because there is no artifact that can overshadow relevant finding.

Moreover, metal stapling appears to be 10 times more expensive than polymer ligation [17].

## 5 Conclusion

As our technique is simple, we believe that Hem-o-lok stapling is feasible for PT during TEP especially for the short type and that it can possibly replace metal stapling. We hope many surgeons who recommend TAPP will consider performing TEP using this technique.

## Ethical approval

None of the authors has nothing to declare.

## Sources of funding

None of the authors has any sources to declare.

## Author contribution

Toshikatsu Nitta is first Author. Takashi Ishibashi is my supervisor and he checked my paper. Jun Kataoka, Masato Ohta, Kesuke Fujii, Yuko Takahashi Ishibashi, They work under my department of SHIROYAMA HOSPITAL. And they engaged in each operations together.

## Trial registry number

We have registered the study with Research Registry (www.researchregistry.com; registration number 5195).

## Guarantor

Takashi Ishibashi ME\D PhD my supervisor Author Toshikatsu Nitta.

## Provenance and peer review

Not commissioned, externally peer reviewed.

## Declaration of competing interest

The authors declare that there is no conflict of interests.
